# Improving Physical Activity among Residents of Affordable Housing: Is Active Design Enough?

**DOI:** 10.3390/ijerph16010151

**Published:** 2019-01-08

**Authors:** Candace Tannis, Araliya Senerat, Malika Garg, Dominique Peters, Sritha Rajupet, Elizabeth Garland

**Affiliations:** 1 Department of Environmental Medicine and Public Health, Icahn School of Medicine at Mount Sinai, New York, NY 10029, USA; asenerat@gmail.com (A.S.); Malika.Garg@mssm.edu (M.G.); dom.peters92@gmail.com (D.P.); Sritha.Rajupet@va.gov (S.R.); Elizabeth.Garland@mssm.edu (E.G.); 2 Department of Primary Care, James J Peters VA Medical Center, Bronx, NY 10468, USA

**Keywords:** active design, physical activity, affordable housing, health behaviors, built environment

## Abstract

Physical inactivity increases risk of chronic disease. Few studies examine how built environment interventions increase physical activity (PA). Active design (AD) utilizes strategies in affordable housing to improve resident health. We assessed how AD housing affects PA among low-income families in Brooklyn, New York. Participants were recruited at lease signings in 2016 from a new AD apartment complex and two recently renovated comparison buildings without AD features. Eligibility included age ≥18 years with no contraindications to exercise. Anthropometric data were collected. PA was self-reported using the Recent and Global Physical Activity Questionnaires. Smartphone users shared their tracked step. Data collection was repeated one year after move-in. All data were analyzed using SPSS. Eighty-eight eligible participants completed the initial questionnaire (36 AD and 52 from 2 comparison buildings) at baseline (T0). There were no differences between AD and comparison cohorts in: stair use, PA, sitting time or, mean waist-to-hip ratio (WHR) at T0. However, the AD cohort had a lower baseline BMI (27.6 vs. 31.0, *p* = 0.019). At one-year follow-up (T1), 75 participants completed our survey including a 64% retention rate among those who previously completed the T0 questionnaire. Among T0 questionnaire respondents, mean daily steps increased at T1 among AD participants who moved from an elevator building (∆6782, *p* = 0.051) and in the comparison group (∆2960, *p* = 0.023). Aggregate moderate work-related activity was higher at T1 in the AD building (746 vs. 401, *p* = 0.031). AD building women reported more work-related PA overall but AD men engaged in more moderate recreational PA. Living in an AD building can enhance low-income residents’ PA. More research with objective measures is needed to identify strategies to sustain higher PA levels and overall health.

## 1. Background

Sedentary behavior and obesity have dramatically increased in the United States overtime [1]. In 2012, the prevalence of obesity among children and adults was 17% and 34.9% respectively in the United States [2]. Inadequate physical activity is a major contributor to the obesity epidemic which is associated with premature mortality and economic loss [3,4].

The U.S. Department of Health and Human Services issued the Physical Activity Guidelines for Americans in 2008 which suggest 60 min of physical activity a day for children and adolescents and at least 150 min of moderate or 75 min of vigorous-intensity exercise for adults that includes at least two or more days of muscle strengthening activities [5]. These guidelines have been shown to improve mental health, control weight, and decrease the risk for chronic medical conditions such as type 2 diabetes, metabolic syndrome, and cardiovascular disease [6].

Despite the plethora of positive effects associated with physical activity, almost 80% of U.S. adults aged 18–64 do not meet the recommended amount of physical activity [7]. Over 30% of adult New Yorkers reported no physical activity in the last 30 days in 2015 [8]. Socioeconomic factors such as lower levels of education and income have negatively impacted physical activity levels amongst these populations while those with more education and higher paying jobs have been found to be more active [9,10]. 

A growing body of evidence supports built environment as a health promoter of individuals and communities by influencing risk factors, such as dietary choice and physical activity. Research has shown individuals living in neighborhoods with more availability of destinations within walking/cycling distance are more likely to engage in physical activity [11].

However, socioeconomically disadvantaged neighborhoods were found to have fewer recreational resources compared to their more affluent counterparts, resulting in higher rates of obesity and diabetes. This trend is especially visible in New York City where the prevalence of obesity and diabetes in the lowest income neighborhoods is at least four times that of the highest income neighborhoods [12]. In addition, perceived safety in a low-income neighborhood may play a role in the reduced usage of neighborhood characteristics, which may indirectly affect physical activity [13]. 

One built environment strategy to improve health outcomes is Active Design (AD): an evidence-based approach to neighborhood development that uses architecture and urban planning to make daily physical activity (PA) and healthy foods more available and inviting. In 2010, the Active Design Guidelines was published in partnerships with several New York City agencies, including the Department of Health and Mental Hygiene and the Department of Design and Construction [14]. This manual is used as a tool to increase physical activity in the design and construction of neighborhoods, streets, and buildings. In AD, building features include: safe bike parking and storage, inviting well-lit stairwells, outdoor fitness equipment, recreation courtyards, and raised garden beds to encourage physical activity [15]. Non-AD buildings tend to follow more traditional construction plans and lack the features described above. 

Literature on neighborhood built environment and youth physical activity show that several environmental features were tied to physical activity levels, including accessibility of spaces and equipment, peer proximity and behavior, adult support or interference, aesthetics, and safety [16]. Furthermore, active transportation within buildings such as use of easily accessible and prominent aesthetically pleasing stairwells have the potential to increase physical activity [17,18,19,20,21]. Other factors that encourage greater stair use include point of decision stair prompts which have been recommended by the Centers for Disease Control and Prevention (CDC) based on strong evidence of their association [22]. 

Despite associations that have been found between different characteristics of the built environment and poor health outcomes related to low PA and obesity, little is known regarding the effect that AD could have on reducing the rates of these outcomes in residential settings [23]. One study that specifically addresses AD in an affordable housing setting showed improvements in BMI, stair use, and percentage of residents achieving the recommended PA goals [21]. Responses from study focus groups also revealed that the AD elements in affordable housing, including the ambiance and feelings of safety, truly supported and promoted the active pursuit of health and wellness [24]. The objective of this project is to evaluate the implementation of AD strategies in affordable housing as a means of increasing PA among low income families and compare to similar housing without AD features. 

## 2. Materials and Methods

### 2.1. Study Setting

A new affordable housing development comprised of five 6-story elevator buildings with 179 units across three adjacent blocks was identified for this study (AD). Occupancy began in April 2016. Active design features include accessible, prominent, and well-lit stairwells with locally-produced artwork; point-of-decision prompts at elevators demonstrating locations of stairs; proposed outdoor community garden areas; outdoor fitness areas at each building with playground facilities for children, and a community gym onsite. Eligibility for housing in the AD building was determined via income criteria based on apartment size and a housing lottery that was publicized to the community through multiple avenues including: newspapers, the internet, and telephone housing hotlines. Current neighborhood residents and civil servant applicants were given priority. For comparison, we identified two apartment complexes in low-income North and Central Brooklyn neighborhoods without the active design features (Non-AD).

### 2.2. Recruitment

Participants were recruited at lease signings in 2016 from AD and two recently renovated, non-AD buildings with a similar socioeconomic background through partnership with building management. Participants were eligible if they were at least 18 years old without contraindications to exercise, were non-pregnant by self-report (women), and were able to communicate in English. Participation in the study was not associated with the apartment rental process which was established prior to the start of the project. Anthropometric data including weight, body mass index (BMI) and waist to hip ratio (WHR) were collected from participants prior to move-in at baseline (T0) by trained research assistants. At T0, physical activity was measured using the Recent Physical Activity Questionnaire and the Global Physical Activity Questionnaire. Smartphone users shared data on the average number of daily steps and flights of stairs climbed over the previous one-month period. The questionnaires are available online as a supplement (Supplementary Tables S1 and S2). Repeat assessments were conducted after participants had resided in their apartments for 12 months (T1) from both the intervention (AD) and controls (Non-AD). T0 data were collected on the same day as study consent whenever possible. All participants signed informed consent forms. The research protocol was approved by the Institutional review board of Icahn School of Medicine at Mount Sinai (ethical code: HS#: 15-01159).

### 2.3. Data Analysis

All data analysis was conducted using SPSS software version 23 (IBM Corp, Armonk, NY, USA). Categorical data were analyzed using Chi-square and McNemar’s tests. Continuous data were analyzed using T-tests and Mann Whitney U test as appropriate. Normality of the data distribution of individual outcome variable was assessed using the Shapiro-Wilk test to identify the appropriate statistical analytic method for each one. 

T0 vs. T1 Comparison:

Descriptive statistics were obtained for both AD and non-AD participants. Univariate analysis was conducted to assess the differences between the two groups and identify potential confounders at T0. 

Individual level changes in anthropometric measures, were assessed at time T0 and T1 using paired T-tests for both groups. Data were stratified by whether the participants previously lived in walk-up building at the time of study enrollment. McNemar’s and chi-square testing was used to assess for change in BMI category, and frequency of stair usage during the week and on weekends. 

Cross Sectional Analyses at T1:

Cross sectional, descriptive analyses were performed at T1 to assess group level differences of anthropometric measures and PA between residents of the AD and the non-AD buildings. The questionnaire responses and anthropomorphic data measures of both groups of participants were compared using t-tests for normally distributed continuous variables, Mann Whitney U tests for non-normally distributed continuous variables, and Chi-square tests for binary data.

## 3. Results

### 3.1. Baseline Demographics

A total of 118 participants were screened for study inclusion at T0, 41 from AD and 67 from the non-AD buildings. Of these, 36 AD building and 52 non-AD participants were eligible, consented and completed the survey at T0. The remaining 5 AD and 15 non-AD individuals did not meet criteria for study enrollment. Reasons for exclusion from the study were: being non-English speaking, having mobility issues as identified through our screening questionnaire, and being visibly pregnant. The majority of enrolled participants were women (76.9% in AD vs. 80.6% non-AD, *p* = 0.864). AD residents were younger and had lower BMI at T0 compared to the non-AD group (34.9 years vs. 41.8 years, *p* = 0.027; 27.6 BMI vs. 31.0 BMI, *p* = 0.019). However, mean WHR was comparable in both groups at baseline (0.831 vs. 0.868, *p* = 0.083). 

Table 1 summarizes demographic data and the responses to the physical activity questionnaires at T0. Only about 30% of individuals in either group reported any work-related vigorous activity. About two thirds of AD participants reported any work-related moderate activity compared to 45% of non-AD participants. Similarly, the AD group was more likely to report recreational activity compared to the non-AD group, but the difference was not statistically significant. Generally, both groups had similar mean minutes of work and recreational PA, and sitting time. However, the non-AD group climbed more flights of stairs per day compared to AD (9.4 vs. 5.0, *p* = 0.032); and the AD group reported more minutes of walking or cycling during the week (717 vs. 450 *p* = 0.019).

### 3.2. One Year Follow-Up

Of those who completed the initial enrollment survey, 21 AD and 35 non-AD participants completed the one-year (T1) assessments which corresponds to 58% and 67% completion rates, respectively. Completion rates were similar in men and women. Of the 15 AD participants who were lost to follow-up, 6 moved away, 7 participants were unreachable, 1 was unable to complete survey due to work schedule conflict and 1 participant died prior to completing the follow-up. In the non-AD group, 17 participants were lost to follow-up. Two participants moved away and the others were unreachable using a combination of telephone, mail, and in-person efforts which were identical in both groups. Those who completed the T1 assessments were an average of 2 years older and had more daily steps and flights climbed at baseline than those who were lost to follow-up. More specific data are available in the Supplementary Materials (Table S3). However, there was no difference in PA questionnaire responses, BMI, or WHR between the two groups.

### 3.3. Individual Participant Level Analyses

Both AD and non-AD building participants reported an increase in mean daily steps, however the increase was greatest among the AD group residents who previously resided in an elevator building. (∆6782, *p* = 0.051), compared to the non-AD group (∆2960, *p* = 0.023) as outlined in Table 2 and Table 3. There was also a statistically significant, greater decline in mean daily flights that was present in the AD residents with prior residence in an elevator building only. (∆−1.5, *p* = 0.05 vs. ∆−1.0, *p* = 0.46). There were no significant changes in self-reported recreational activity, time spent walking/cycling for travel, or sitting time, in either participant group, or within the sub-group analysis of AD building residents who previously resided in an elevator building. There was no difference in BMI category shift, or change in weekday and weekend stair use frequency between the two groups. 

Work related moderate activity minutes per week declined from T0 to T1 in both groups, with a larger decline in the AD group (∆−705, *p* = 0.027; ∆−436, *p* = 0.016). Mean BMI increased in the AD building but not in the non-AD building (∆1.46, *p* = 0.007; ∆−0.17, *p* = 0.633). WHR remained stable in both groups at follow-up. 

### 3.4. Secondary Cross-Sectional Responses 

Seventy-five participants completed the survey at T1, 27 from AD and 48 from our non-AD buildings, including the 21 AD and 35 non-AD building residents who also completed the T0 questionnaire. Six AD and 13 non-AD building participants who were not enrolled at T0 also completed the T1 questionnaire. The majority of T1 survey respondents were women (81.5% in AD and 68.7% non-AD, *p* = 0.707). Among all participants who completed the T1 survey, BMI and WHR were comparable in the two groups (30.9 vs. 31.6, *p* = 0.671, 0.881 vs. 0.847, *p* = 0.244). The majority of participants in both groups lived above the ground floors of their building at the time of survey (74.1% and 81.3%). However, non-AD building residents were less likely to live on the ground floor (4.2% vs. 22.2%, *p* = 0.05). Data on floor of residence were unavailable for 3.7% (*n* = 1) of AD and 14.5% (*n* = 7) of non-AD participants. Table 4 summarizes the T1 demographic information, as well as BMI and WHR stratified by gender, age, and whether participants completed the survey at T0. Data on daily steps and flights of stairs climbed were also stratified by whether or not the residents lived on a ground floor apartment. Our data showed no differences in questionnaire responses between AD and the non-AD buildings. However, among newly enrolled T1 participants, WHR was higher in the AD group (0.93 vs. 0.81, *p* = 0.03). 

Participants who lived in the non-AD buildings reported more daily steps taken and flights stairs climbed compared to AD residents (5483 vs. 9151, *p* = 0.001; 3.8 vs. 7.2, *p* = 0.022) as outlined in Table 5. These trends persisted in our subgroup analyses of participants who were enrolled at T0 and new participants, as well as after stratification by apartment type. Interestingly, new non-AD building participants spent more than twice as much time sitting compared to their AD counterparts (275 min vs. 112 min, *p* = 0.022). 

AD men reported more moderate recreational activity minutes (1115 vs. 131, *p* = 0.044), but there were no significant differences among women. On the contrary, women who resided in the AD building reported more work-related vigorous and moderate activity minutes (404 vs. 32, *p* = 0.01; 865 vs. 371, *p* = 0.015), but no differences were found among men. Aggregate moderate work-related activity minutes were also higher in the AD group than in the non-AD group among previously enrolled participants despite the individual level declines that were discussed earlier (746 vs. 401, *p* = 0.031).

Differences in questionnaire responses stratified by age category were analyzed. Among older participants (age ≥ 50 years), those in AD were generally more active than those in the non-AD building including a statistically significant difference in weekly recreational moderate activity (1034 vs. 72 min, *p* = 0.029). Younger non-AD participants (age < 50 years) reported more than twice as many daily flights climbed as those residing in AD (7.8 vs. 3.3, *p* = 0.017). There were no other between-group differences in this subpopulation. 

## 4. Discussion

We found that residing in an active design (AD) building can positively impact residents’ stair usage. Our findings are consistent with previous literature that AD design strategies that target stair use can positively impact behavior change [25]. One study found that residents of buildings with active design components were significantly more likely to have reported increased stair use and less likely to have reported no change or decreased stair use than residents who did not [21]. Overall, we found that residents in our non-AD buildings had higher levels of objectively measured PA which highlights the multifactorial nature of PA-related health behaviors. A study by Zimmerman et al. found that poorer self-rated health and factors like smoking were predictors of decreased PA [26]. It is possible that our non-AD building residents had higher self-rating of their health which affected their self-efficacy for using the stairs and being more active during the day. In addition, the non-AD building residents had a stronger sense of community and better relationship with building staff which may translate to increase likelihood of pursuing more PA in the neighborhood. This is consistent with previous literature showing that residents with smaller social networks were less physically active than those with larger social networks after adjusting for employment status, gender, poverty level, current health status, age, and perceived neighborhood safety [27]. Although the overall work-associated and recreational PA were higher in the AD group at baseline and at T1, the differences were not statistically significant. Also, these data were self-reported which is an important limitation. Research shows that individuals tend to overestimate their PA which can contribute to the energy imbalance that leads to weight gain [28]. 

Women who lived in the AD building at T1 reported more moderate and vigorous work-related physical activity but the same trend was not noted for recreational physical activity. One possible explanation is that people who are more active at work may be less willing to engage in recreational physical activity. A 2004 study found that engaging in high levels of occupational PA was associated with more transport activity but less leisure time PA [29]. Furthermore, we did not assess participants’ occupation or characteristics of the work environment. It is possible that the participants’ work environments or neighborhoods of employment were more supportive of physical activity behaviors. This is consistent with literature suggesting that workplace built environment features such as presence of shops, bike and recreation facilities, and reduced crime rate can increase workplace PA [30].

Based on our data on gender differences in physical activity, AD women were more likely to be active compared to women in the non-AD building. However, those differences were not observed among men. Previous research has shown women report lower levels of physical activity than men, but the differences may be due to the types of activities they participate in rather than the true differences in activity levels [31]. Household work, child care, and other caregiving physical activity are reported more frequently by women compared to men, who report more sports and leisure-time exercise [31]. Predictors of physical activity also vary among women and include age, social roles, social support, and structural/environmental variables. Women in the non-AD building may have more social support and a closer community, which may serve as a predictor of exercise levels. Lack of information regarding social support and other socio-economic factors is a limitation in this study.

This study showed younger non-AD participants were more likely to use the stairs than younger AD participants, but older AD participants reported more recreational moderate physical activity than older non-AD participants. This is similar to what has been seen in previous research, where activities that require a higher exertion level decline consistently as individuals age; and relatively stable physical activity patterns appear in middle to late adulthood [32]. Older adults may take part in physical activity because of more concern for physical and psychological health issues, compared to younger participants who participate in sports for play [33]. Results from this study regarding age differences are not surprising, but lack of qualitative data is a limitation. Future studies should consider collecting qualitative data from younger and older participants regarding reasons for physical activity or lack thereof.

We found an increased BMI among AD residents at T1 and stable BMI among the non-AD building residents, as well as stable WHR in both groups during the same time period. These results are consistent with previous literature suggesting that BMI is impacted by factors other than physical activity. A 2006 Cochrane review showed that physical activity combined with diet resulted in a greater weight reduction than diet alone, with higher intensity resulting in a larger magnitude of weight loss [34]. However, we did not assess diet quality or changes in dietary patterns during our study which may have impacted our BMI and WHR findings. 

Our study does have some limitations, such as the use of a non-random sample of participants which may have contributed to our study population being predominantly women with a noticeable age difference between the two groups at baseline. The lottery process used to identify potential residents for AD could have selected for individuals seeking an AD apartment resulting in a younger pool of applicants who were interested in the types of amenities that were offered. Stable employment was another criterion used for AD applicant screening that may have contributed to study bias. Though we were able to control for seasonal variation in PA by ensuring that our T1 follow-up data was collected during the same time of year as baseline, we were unable to account for differences in weather patterns that may have impacted participants’ self-reported PA within the past week which was the time frame of the PA questions. Our follow-up surveys were scheduled at the convenience of building staff based on their availability to grant us access. As a result, most of our follow-ups were conducted in the late afternoons and early evening on weekdays. This may have prevented some working individuals from being able to complete our one-year assessments and contribute to our loss to follow-up, since the assessments needed to be conducted in person. Individuals who are employed regularly tend to be healthier than those who are not [35]. We hypothesize that since the AD building participants were younger, differential loss to follow-up due to lack of availability and competing priorities may have resulted in an underestimate of AD benefits. Our study sample was small due to recruitment challenges associated with varying level of participation interest based on competing priorities of both the real estate management and potential tenants. We acknowledge the skewed distribution of our PA outcome variables which could be addressed in follow-up studies by increasing the sample size and using additional objective measures to PA to reduce reporting bias. We also did not assess other socioeconomic variables that may also contribute to physical inactivity. Nevertheless, our study adds to the growing body of literature using community-based data collection methods which is important for assessing the impact of health interventions such as AD housing in a real-world setting. 

## 5. Conclusions

Many low-income and minority families live in neighborhoods that are not designed to support physical activity and healthy eating, which contributes to these populations being disproportionately affected by chronic disease. Our finding that moving from a non-AD elevator building to an AD elevator building can positively impact daily steps demonstrates the potential of affordable housing environments to improve health among those who are most affected by health disparities related to exposure to an unhealthy environment. Broad adoption of affordable housing AD strategies is warranted to encourage behavior change that improves physical activity measures and is consistent with the Community Preventive Services Task Force recommendations for community engagement around this important lifestyle modification [36]. Additional research to identify health behavior and community-based interventions that can be implemented among affordable housing residents to supplement active design housing features is also important for informing stakeholders and public health advocates looking to promote health outcomes for neighborhoods in need. 

## Figures and Tables

**Table 1:** Baseline (T0) Demographic Data and Physical Activity Level.

	AD (*n* = 36)	Non-AD (*n* = 52)	*p*-Value
Body Mass Index (BMI)	27.6 (SD = 5.08)	31.0 (SD = 8.35)	0.019
Waist-to-hip Ratio (WHR)	0.84 (SD = 0.09)	0.87 (SD = 0.10)	0.859
Age	34.9 (SD = 14.50)	41.8 (SD = 13.20)	0.03
Gender (% Men)	19.4% (*n* = 7)	23.1% (*n* = 12)	0.684
Work VPA (%)	31.4% (*n* = 11)	28.6% (*n* = 14)	0.482
Mean minutes per week ^a^	513 (SD = 1126.7)	467(SD = 995.7)	0.881
Work MPA (%)	65.7% (*n* = 23)	44.9% (*n* = 22)	0.059
Mean minutes per week ^a^	887 (SD = 1065.6)	749 (SD = 1136.7)	0.154
Walk/cycle for travel (%)	88.5% (*n* = 31)	77.6% (*n* = 38)	0.194
Mean minutes per week ^a^	717 (SD = 843.9)	450 (SD = 1097.8)	0.019 *
Recreational VPA activity (%)	54.2% (*n* = 19)	33.3% (*n* = 6)	0.056
Mean minutes per week ^a^	202 (SD = 312.8)	113 (SD = 241.9)	0.078
Recreational MPA activity (%)	50% (*n* = 18)	43.7% (*n* = 21)	0.489
Mean minutes per week ^a^	218 (SD = 410.2)	102 (SD = 195.7)	0.138
Daily sitting minutes	272 (SD = 203.8)	245 (SD = 196.2)	0.591
Steps per day	4573 (SD = 3038.7)	5388 (SD = 3378.3)	0.542
Stair flights per day ^a^	5.0 (SD = 2.6)	9.4 (SD = 5.3)	0.03 *

^a^ Denotes a non-normal distributed variable with significant skew. Note: Means and medians were calculated but means are displayed for consistency due to large number of individuals with no reported physical activity. AD = Active Design. BMI = body mass index. WHR = waist-to-hip ratio. Work VPA % = % of individuals reporting any vigorous activity at work. Work MPA % = % of individuals reporting any vigorous activity at work. Recreational VPA % = % of individuals reporting any recreational vigorous activity. Recreational MPA % = % of individuals reporting any moderate recreational activity. * Denotes statistically significant value.

**Table 2:** Pre-Post Paired Comparisons for Anthropometric and Physical Activity Response Variables.

Variable	Non-AD Δ (SD) (*n* = 35)	AD Δ (SD) (*n* = 21)
Δ BMI	−0.17 (SD = 2.04)	1.46 (SD = 2.14)
*p-value*	0.633	0.007
BMI category shift (%) ^a^		
Category Decrease	8.6%	5%
No change	80%	60%
Category Increase	11.4%	35%
*p-value*	0.058	0.058
Δ WHR	0.001 (SD = 0.11)	0.045 (SD = 0.16)
*p-value*	0.997	0.212
Δ Recreational VPA mins per week ^b^	−24 (SD = 232.2)	−83 (SD = 241.4)
*p-value*	0.522	0.131
Δ Recreational MPA mins per week ^b^	2 (SD = 244.5)	64 (SD = 1123.8)
*p-value*	0.963	0.795
Δ Sitting mins per day ^b^	26(SD = 176.8)	11 (SD = 302.2)
*p-value*	0.413	0.867
Δ Stair flights per day ^b^	−1.0 (SD = 2.7)	−1.0 (SD = 1.4)
*p-value*	0.46	0.252
Δ Steps per day	2960 (SD = 2233.2)	3607 (SD = 5431.5)
*p-value*	0.023 *	0.212
∆ Weekday stair use frequency (%) ^a^		
Category Decrease	21.2%	19%
No change	45.5%	52.4%
Category Increase	33.2%	28.6%
*p-value*	0.897	0.897
∆ Weekend stair use frequency shift (%) ^a^		
Category Decrease	12.5%	35%
No change	53.1%	40%
Category Increase	34.4%	25%
*p-value*	0.121	0.121
Δ Work VPA mins per week ^b^	−206 (SD = 594.5)	−216 (SD = 953.6)
*p-value*	0.059	0.311
Δ Work MPA mins per week ^b^	−436 (SD = 970.4)	−705 (SD = 1353.8)
*p-value*	0.016 *	0.027 *
Δ Walk/cycle mins per week ^b^	−141 (SD = 1443.4)	−8 (SD = 1148.5)
*p-value*	0.578	0.974

^a^ Denotes % of participants. Note: Means and medians were calculated but means are displayed for consistency due to large number of individuals with no reported physical activity. ^b^ Denotes a non-normal distributed variable with significant skew. Category change is based on scale from lower to higher BMI and from lower to higher steps/stair flight frequency. See survey in Supplementary Materials. VPA = Vigorous physical activity; MPA = Moderate physical activity. * Denotes statistically significant result.

**Table 3:** Sub-group analyses of AD Building Anthropometric and Physical Activity Response Variables.

Variable	AD Elevator Only Δ (SD) (*n* = 11)	AD Prior Walk-up Only Δ (SD) (*n* = 10)
Δ BMI	0.59 (SD = 1.61)	2.52 (SD = 2.31)
*p-value*	0.254	0.011
BMI category shift (%) ^a^		
Category Decrease	N/A	N/A
No change	N/A	N/A
Category Increase	N/A	N/A
*p-value*	N/A	N/A
Δ WHR	0.003 (SD = 0.07)	0.010 (SD = 0.23)
*p-value*	0.864	0.22
Δ Recreational VPA mins per week ^b^	−40 (SD = 153.0)	−140 (SD = 327.3)
*p-value*	0.385	0.235
Δ Recreational MPA mins per week ^b^	274 (SD = 1375.5)	−216 (SD = 633.7)
*p-value*	0.504	0.337
Δ Sitting mins per day ^b^	71.(SD = 312.0)	−69 (SD = 286.3)
*p-value*	0.446	0.491
Δ Stair flights per day ^b^	−1.5 (SD = 2.1)	−1.7 (SD=2.0)
*p-value*	0.05 *	0.30
Δ Steps per day	6783 (SD = 2760.8)	1157(SD = 5207.1)
*p-value*	0.051 *	0.806
∆Weekday stair use frequency (%) ^a^		
Category Decrease	N/A	N/A
No change	N/A	N/A
Category Increase	N/A	N/A
*p-value*	N/A	N/A
∆ Weekend stair use frequency shift (%) ^a^		
Category Decrease	N/A	N/A
No change	N/A	N/A
Category Increase	N/A	N/A
*p-value*	N/A	N/A
Δ Work VPA mins per week ^b^	−54 (SD = 597.5)	−433 (SD = 1300.0)
*p-value*	0.761	0.347
Δ Work MPA mins per week ^b^	−703 (SD = 1386.4)	−708 (SD = 1392.6)
*p-value*	0.107	0.166
Δ Walk/cycle mins per week ^b^	−223 (SD = 1332.9)	278 (SD = 832.4)
*p-value*	0.573	0.345

^a^ Denotes % of participants. Note: Means and medians were calculated but means are displayed for consistency due to large number of individuals with no reported physical activity. ^b^ Denotes a non-normal distributed variable with significant skew. Category change is based on scale from lower to higher BMI; and from lower to higher steps/stair flight frequency. See survey in Supplementary Materials. VPA = Vigorous physical activity MPA = Moderate physical activity. * Denotes statistically or borderline statistically significant result.

**Table 4:** Stratified Cross-sectional One Year (T1) Anthropometric and Demographic Data.

Variable	AD Building (*n* = 27)	Non-AD Building (*n* = 48)	*p*-Value
Sex			0.707
Women (%)	81.5% (*n* = 22)	68.7% (*n* = 33)	
Men (%)	18.5% (*n* = 5)	31.3% (*n* = 15)	
Apartment Type			0.05 *
Ground Floor (%)	22.2% (*n* = 6)	4.2% (*n* = 2)	
Above Ground Floor (%)	74.1% (*n* = 20)	81.3% (*n* = 39)	
Unknown	3.7% (n = 1)	14.5% (*n* = 7)	
Enrolled at Baseline			0.59
Yes (%)	77.8% (*n* = 21)	72.9% (*n* = 35)	
No (%)	22.2% (*n* = 6)	27.1% (*n* = 13)	
Age			
All Participants	35.7 (SD = 14.56)	39.9 (SD = 14.01)	0.154
Previously Enrolled	34.9 (SD = 14.50)	41.8 (SD = 13.20)	0.027
Not Previously Enrolled	42.0 (SD = 15.60)	31.2 (SD = 14.97)	0.242
Women	33.4 (SD = 12.13)	41.2 (SD = 14.23)	0.018 *
Men	37.7 (SD = 13.28)	36.5 (SD = 13.23)	0.828
Body Mass Index (BMI)			
All Participants	30.8 (SD = 6.72)	31.6 (SD = 7.90)	0.671
Women	30.5 (SD = 1.54)	32.3 (SD = 8.80)	0.438
Men	32.2 (SD = 3.86)	30.0 (SD = 5.12)	0.389
Age < 50	30.8 (SD = 7.50)	31.7 (SD = 8.40)	0.713
Age ≥ 50	29.6 (SD = 4.25)	32.5 (SD = 4.76)	0.266
Previously Enrolled	29.9 (SD = 7.08)	31.6 (SD = 8.41)	0.444
Not Previously Enrolled	34.1 (SD = 4.29)	31.6 (SD = 6.54)	0.42
Waist to Hip Ratio (WHR)			
All Participants	0.88 (SD = 0.15)	0.85 (SD = 0.11)	0.244
Women	0.87 (SD = 0.15)	0.83 (SD = 0.10)	0.249
Men	0.92 (SD = 0.10)	0.88 (SD = 0.13)	0.479
Age < 50	0.86 (SD = 0.16)	0.85 (SD = 0.11)	0.631
Age ≥ 50	0.90 (SD = 0.09)	0.86 (SD = 0.12)	0.574
Previously Enrolled	0.87 (SD = 0.15)	0.86 (SD = 0.11)	0.814
Not Previously Enrolled	0.93 (SD = 0.12)	0.81 (SD = 0.09)	0.03 *

Note: Means and medians were calculated but means are displayed for consistency due to large number of individuals with no reported physical activity resulting in a median value of zero. * Denotes statistically significant result.

**Table 5:** Stratified Cross-sectional One Year (T1) Physical Activity Questionnaire Responses.

Variable	AD Building (*n* = 27)	Non-AD Building (*n* = 48)	*p*-Value
Recreational VPA mins per week ^a^			
Any (% Yes)	33.3% (*n* = 9)	41.7% (*n* = 20)	0.392
All Participants	189 (SD = 644.4)	156 (SD = 307.4)	0.802
Women	222 (SD = 712.1)	113 (SD = 253.3)	0.71
Men	48 (SD = 78.2)	270 (SD = 408.1)	0.391
Age < 50	50 (SD = 111.3)	157 (SD = 301.7)	0.243
Age ≥ 50	98 (SD = 157.0)	3 (SD = 10.0)	0.456
Previously Enrolled	61 (SD = 124.6)	64 (SD = 160.7)	0.694
Not Previously Enrolled	638 (SD = 1337.0)	427 (SD = 455.7)	0.521
Recreational MPA mins per week ^a^			
Any (% Yes)	48.1% (*n* = 13)	50% (*n* = 24)	0.67
All Participants	335 (SD = 861.2)	98 (SD = 142.6)	0.81
Women	158 (SD = 397.0)	84 (SD = 125.8)	0.62
Men	1115 (SD = 1749.4)	131 (SD = 175.9)	0.044 *
Age <50	143 (SD = 397.0)	110 (SD = 140.7)	0.223
Age ≥ 50	1034 (SD = 1598.6)	72 (SD = 166.3)	0.029 *
Previously Enrolled	331 (SD = 959.7)	92 (SD = 148.7)	0.945
Not Previously Enrolled	350 (SD = 415.1)	115 (SD = 129.9)	0.416
Work VPA mins per week ^a^			
Any (% Yes)	40.7% (*n* = 11)	20.8% (*n* = 10)	0.083
All Participants	601 (SD = 1076.1)	227 (SD = 547.5)	0.102
Women	404 (SD = 865.7)	32 (SD = 127.5)	0.01 *
Men	1470 (SD = 1560.3)	644 (SD = 822.1)	0.395
Age < 50	308 (SD = 653.8)	224 (SD = 546.8)	0.596
Age ≥ 50	620 (SD = 930.8)	75 (SD = 212.1)	0.228
Previously Enrolled	229 (SD = 566.5)	130 (SD = 421.5)	0.388
Not Previously Enrolled	1905 (SD = 1454.4)	480 (SD = 751.4)	0.036 *
Work MPA mins per week ^a^			
Any (% Yes)	66.7% (*n* = 18)	35.4% (*n* = 17)	0.011 *
All Participants	912 (SD = 1131.5)	464 (SD = 845.5)	0.022 *
Women	865 (SD = 1180.1)	371 (SD = 885.1)	0.015 *
Men	1116 (SD = 972.7)	664 (SD = 742.7)	0.395
Age < 50	529 (SD = 790.6)	426 (SD = 719.8)	0.322
Age ≥ 50	1545 (SD =1392.4)	503 (SD = 1327.0)	0.059
Previously Enrolled	746 (SD = 1066.0)	401 (SD = 863.8)	0.031 *
Not Previously Enrolled	1490(SD = 1263.4)	631 (SD = 804.3)	0.152
Walking/Cycling mins per week ^a^			
Any (% Yes)	88.9% (*n* = 24)	87.5% (*n* = 42)	0.95
All Participants	830 (SD = 1062.3)	478 (SD = 561.0)	0.245
Women	622 (SD =773.5)	558 (SD = 2.2)	0.798
Men	1745 (SD = 1705.9)	306 (SD = 312.2)	0.098
Age < 50	510 (SD = 607.0)	476 (SD = 618.0)	0.957
Age ≥ 50	1809 (SD = 1647.0)	318 (SD = 257.0)	0.059
Previously Enrolled	665 (SD = 892.1)	420 (SD = 497.8)	0.40
Not Previously Enrolled	1408 (SD = 1472.8)	628 (SD = 701.1)	0.179
Daily Sitting mins per day ^a^			
All Participants	256 (SD =202.5)	267 (SD = 182.6)	0.816
Women	259 (SD = 203.6)	272.(SD = 204.0)	0.829
Men	240(SD = 220.5)	255(SD = 126.6)	0.854
Age < 50	292 (SD = 204.9)	280 (SD = 181.7)	0.824
Age ≥ 50	222 (SD = 212.1)	220 (SD = 196.5)	0.988
Previously Enrolled	297 (SD = 206.7)	263(SD = 200.2)	0.504
Not Previously Enrolled	112 (SD = 101.3)	275 (SD = 134.6)	0.022 *
Mean Daily Steps			
All Participants	5483 (SD = 2396.7)	9151 (SD = 2470.8)	0.001 *
Women	4928 (SD = 2231.0)	8598 (SD = 2351.7)	0.012
Men	6315 (SD = 2719.7)	9889 (SD = 2643.4)	0.072
Age < 50	5092 (SD = 2061.4)	9472 (SD = 2477.4)	0.002
Age ≥ 50	6165 (SD = 3745.1)	5958 (N/A)	0.966
Previously Enrolled	6169 (SD = 2739.3)	8748(SD = 2384.2)	0.075
Not Previously Enrolled	4454 (SD = 1540.1)	9877 (SD = 2729.3)	0.01 *
Ground Floor Apt	8296 (SD = 2580.2)	11000 (N/A)	0.549
Above Ground Floor Apt	4637 (SD = 2004.7)	9051 (SD = 2618.2)	0.001 *
Mean Daily Flights ^a^			
All Participants	3.8 (SD = 3.0)	7.2 (SD = 3.0)	0.022 *
Women	3.2 (SD = 3.6)	6.6 (SD = 2.2)	0.093
Men	5.0 (SD = 1.0)	7.7 (SD = 3.7)	0.276
Age < 50	3.3 (SD = 3.6)	7.8 (SD = 2.8)	0.017 *
Age ≥ 50	4.0 (SD = 1.4)	2.0 (N/A)	0.454
Previously Enrolled	3.2 (SD = 4.0)	6.5 (SD = 3.7)	0.189
Not Previously Enrolled	4.5 (SD = 1.3)	8.0 (SD = 2.0)	0.02 *
Ground Floor Apt	1.0 (N/A)	N/A	N/A
Above Ground Floor Apt	3.9 (SD = 3.1)	7.2 (SD = 3.0)	0.049 *

^a^ Denotes a non-normal distributed variable with significant skew. Note: Means and medians were calculated but only means are displayed due to large number of individuals with no reported physical activity. VA = Vigorous activity MA = Moderate activity. * Denotes statistically significant result.

## Supplementary Materials

The following are available online at http://www.mdpi.com/1660-4601/16/1/151/s1, Table S1. Recent Physical Activity Questionnaire (RPAQ); Table S2. Global Physical Activity Questionnaire (GPAQ); Table S3: Stratification of Baseline by Survey Completion at T1.

## References

[1] Centers for Disease Control and Prevention National Center for Chronic Disease Prevention and Health Promotion, Division of Nutrition, Physical Activity, and Obesity. Data, Trend and Maps. https://www.cdc.gov/nccdphp/dnpao/data-trends-maps/index.html.

[2] Ogden C.L., Carroll M.D., Kit B.K., Flegal K.M. (2014). Prevalence of Childhood and Adult Obesity in the United States, 2011–2012. JAMA.

[3] Blair S.N. (2009). Physical inactivity: The biggest public health problem of the 21st century. Br. J. Sports Med..

[4] Tremmel M., Gerdtham U.G., Nilsson P.M., Saha S. (2017). Economic Burden of Obesity: A Systematic Literature Review. Int. J. Environ. Res. Public Health.

[5] 2008 Physical Activity Guidelines for Americans. https://health.gov/paguidelines/guidelines/summary.aspx.

[6] 2018 Physical Activity Guidelines Advisory Committee (2018). 2018 Physical Activity Guidelines Advisory Committee Scientific Report.

[7] Blackwell D.L., Clarke T.C. (2018). State Variation in Meeting the 2008 Federal Guidelines for Both Aerobic and Muscle-Strengthening Activities through Leisure-Time Physical Activity among Adults Aged 18–64: United States 2010–2015.

[8] Hypertension in New York City: Disparities in Prevalence. https://www1.nyc.gov/assets/doh/downloads/pdf/epi/databrief82.pdf.

[9] Shuval K., Li Q., Gabriel K.P., Tchernis R. (2017). Income, physical activity, sedentary behavior, and the ‘weekend warrior’ among U.S. adults. Prev. Med..

[10] QuickStats: Percentage of Adults Aged >25 Years Who Reported Regular Leisure-Time Physical Activity, by Education Level—National Health Interview Survey, United States, 1997 and 2007. https://www.cdc.gov/mmwr/preview/mmwrhtml/mm5810a7.htm.

[11] Zapata-Diomedi B., Veerman J.L. (2016). The association between built environment features and physical activity in the Australian context: A synthesis of the literature. BMC Public Health.

[12] Community Health Survey Atlas 2015. https://www1.nyc.gov/assets/doh/downloads/pdf/data/2015_CHP_Atlas.pdf.

[13] Foster S., Giles-Corti B. (2008). The built environment, neighborhood crime and constrained physical activity: An exploration of inconsistent findings. Prev. Med..

[14] Lee K.K. (2012). Developing and implementing the Active Design Guidelines in New York City. Health Place.

[15] Center for Active Design Active Design Guidelines: Promoting Physical Activity and Health in Design. https://centerforactivedesign.org/guidelines/.

[16] Loman D.G. (2008). Promoting physical activity in teen girls: Insight from focus groups. MCN Am. J. Matern. Child Nurs..

[17] Troiano R.P., Berrigan D., Dodd K.W., Masse L.C., Tilert T., McDowell M. (2008). Physical activity in the United States measured by accelerometer. Med. Sci. Sports Exerc..

[18] Nicoll G. (2007). Spatial measures associated with stair use. Am. J. Health Promot..

[19] Boutelle K.N., Jeffery R.W., Murray D.M., Schmitz M.K. (2001). Using signs, artwork, and music to promote stair use in a public building. AJPH.

[20] Kerr N.A., Yore M.M., Ham S.A., Dietz W.H. (2004). Increasing stair use in a worksite through environmental changes. Am. J. Health Promot..

[21] Garland E., Garland V., Peters D., Doucette J., Thanik E., Rajupet S., Sanchez S. (2018). Active design in affordable housing: A public health nudge. Prev. Med. Rep..

[22] Task Force on Community Preventive Services (2002). The effectiveness of interventions to increase physical activity. Prev. Med..

[23] Chamber E.C., Fuster D. (2012). Housing as an obesity-mediating environment. Int. J. Public Health.

[24] Garland E., Baban K., Garland V., Bey G., Sanchez S. (2014). One Step at a Time Towards Better Health: Active Design in Affordable Housing. Environ. Justice.

[25] Boarnet M.G., Handy S.L., Ewing R., Lillingsworth R.E. (2002). How the built environment affects physical activity: Views from urban planning. Prev. Med..

[26] Zimmermann E., Ekholm O., Grønbæk M., Curtis T. (2008). Predictors of changes in physical activity in a prospective cohort study of the Danish adult population. Scand. J. Public Health.

[27] Shelton R.C., McNeill L.H., Puleo E., Wolin K.Y., Emmons K.M., Bennett G.G. (2011). The Association between Social Factors and Physical Activity Among Low-Income Adults Living in Public Housing. AJPH.

[28] Physical Activity and Transit Survey: Summary Data. https://www1.nyc.gov/assets/doh/downloads/pdf/epi/PAT-survey-summary.pdf.

[29] Macintyre S., Mutrie N. (2004). Socio-economic differences in cardiovascular disease and physical activity: Stereotypes and reality. J. R. Soc. Promot. Health.

[30] Adlakha D., Hipp A.J., Marx C., Yang L., Tabak R., Dodson E.A., Brownson R.C. (2015). Home and Workplace Built Environment Supports for Physical Activity. Prev. Med..

[31] Belza B., Warms C. (2004). Physical activity and exercise in women’s health. Nurs. Clin. N. Am..

[32] Caspersen C.J., Pereira M.A., Curran K.M. (2000). Changes in physical activity patterns in the United States, by sex and cross-sectional age. Med. Sci. Sports Exerc..

[33] Molanorouzi K., Khoo S., Morris T. (2015). Motives for adult participation in physical activity: Type of activity, age, and gender. BMC Public Health.

[34] Shaw K.A., Gennat H.C., O’Rourke P., Del Mar C. (2006). Exercise for overweight or obesity. Cochrane Database Syst. Rev..

[35] Worklessness and Health—What do we Know about the Causal Relationship?. http://www.employabilityinscotland.com/media/83147/worklessness-and-health-what-do-we-know-about-the-relationship.pdf.

[36] Task Force on Community Preventive Services (2002). Recommendations to increase physical activity in communities. Prev. Med..

