# Supplementary material for: Improving Physical Activity among Residents of Affordable Housing: Is Active Design Enough?
Source: Int J Environ Res Public Health. 2019 Jan 8;16(1):151. doi: 10.3390/ijerph16010151 (PMC6338942; doi:10.3390/ijerph16010151)
Supplement: Supplementary file 1 [file ijerph-16-00151-s001.pdf]

International Journal of
*Environmental Research and Public Health*

MDPI

**Table S1.** Recent Physical Activity Questionnaire.

# Recent Physical Activity Questionnare (RPAQ)

Do you currently live in walk-up building?...............YES/NO
Does your building have a working elevator most days of the week?..........YES/NO
If no, explain_____________________

**Stair climbing at home** (please put a tick (✓) on every line)

| Number of times you climbed up a flight of stairs (approx 10 steps) each day at home | **Average over the last 4 weeks** | | | | | |
|---|---|---|---|---|---|---|
| | None | 1 to 5 times a day | 6 to 10 times a day | 11 to 15 times a day | 16 to 20 times a day | More than 20 times a day |
| On a weekday | | | | | | |
| On a weekend day | | | | | | |

## Smartphone Data

| DATE (for the last month) | # of stairs climbed (in flights) | # of steps taken |
|---|---|---|
| *mm/dd/yy* | | |
| | | |
| | | |
| | | |
| | | |
| | | |
| | | |
| | | |
| | | |
| | | |
| | | |

## Body Measurements

| **Height (cm)** | **Weight (cm)** | **Body fat (%)** | **Waist (cm)** | **Hip (cm)** |
|---|---|---|---|---|
| | | | | |

**Table S2.** Global Physical Activity Questionnaire.

| Physical Activity, Continued | | |
|---|---|---|
| **Question** | **Response** | **Code** |
| **Recreational activities** | | |
| The next questions exclude the work and transport activities that you have already mentioned.<br>Now I would like to ask you about sports, fitness and recreational activities (leisure), *[insert relevant terms]*. | | |
| Do you do any vigorous-intensity sports, fitness or recreational *(leisure)* activities that cause large increases in breathing or heart rate like *[running or football]* for at least 10 minutes continuously?<br>*[INSERT EXAMPLES] (USE SHOWCARD)* | Yes 1<br>No 2 *If No, go to P 13* | P10 |
| In a typical week, on how many days do you do vigorous-intensity sports, fitness or recreational *(leisure)* activities? | Number of days └─┘ | P11 |
| How much time do you spend doing vigorous-intensity sports, fitness or recreational activities on a typical day? | Hours : minutes └─┴─┘ : └─┴─┘ hrs mins | P12 (a-b) |
| Do you do any moderate-intensity sports, fitness or recreational *(leisure)* activities that cause a small increase in breathing or heart rate such as brisk walking, *[cycling, swimming, volleyball]* for at least 10 minutes continuously?<br>*[INSERT EXAMPLES] (USE SHOWCARD)* | Yes 1<br>No 2 *If No, go to P16* | P13 |
| In a typical week, on how many days do you do moderate-intensity sports, fitness or recreational *(leisure)* activities? | Number of days └─┘ | P14 |
| How much time do you spend doing moderate-intensity sports, fitness or recreational *(leisure)* activities on a typical day? | Hours : minutes └─┴─┘ : └─┴─┘ hrs mins | P15 (a-b) |
| **Sedentary behaviour** | | |
| The following question is about sitting or reclining at work, at home, getting to and from places, or with friends including time spent sitting at a desk, sitting with friends, traveling in car, bus, train, reading, playing cards or watching television, but do not include time spent sleeping.<br>*[INSERT EXAMPLES] (USE SHOWCARD)* | | |
| How much time do you usually spend sitting or reclining on a typical day? | Hours : minutes └─┴─┘ : └─┴─┘ hrs mins | P16 (a-b) |

| **Physical Activity** | | |
|---|---|---|
| Next I am going to ask you about the time you spend doing different types of physical activity in a typical week. Please answer these questions even if you do not consider yourself to be a physically active person.<br>Think first about the time you spend doing work. Think of work as the things that you have to do such as paid or unpaid work, study/training, household chores, harvesting food/crops, fishing or hunting for food, seeking employment. *[Insert other examples if needed].* In answering the following questions 'vigorous-intensity activities' are activities that require hard physical effort and cause large increases in breathing or heart rate, 'moderate-intensity activities' are activities that require moderate physical effort and cause small increases in breathing or heart rate. | | |
| **Question** | **Response** | **Code** |
| **Work** | | |
| Does your work involve vigorous-intensity activity that causes large increases in breathing or heart rate like *[carrying or lifting heavy loads, digging or construction work]* for at least 10 minutes continuously?<br>*[INSERT EXAMPLES] (USE SHOWCARD)* | Yes 1<br>No 2 *If No, go to P 4* | P1 |
| In a typical week, on how many days do you do vigorous-intensity activities as part of your work? | Number of days └─┘ | P2 |
| How much time do you spend doing vigorous-intensity activities at work on a typical day? | Hours : minutes └─┴─┘ : └─┴─┘<br>hrs mins | P3 (a-b) |
| Does your work involve moderate-intensity activity, that causes small increases in breathing or heart rate such as brisk walking *[or carrying light loads]* for at least 10 minutes continuously?<br>*[INSERT EXAMPLES] (USE SHOWCARD)* | Yes 1<br>No 2 *If No, go to P 7* | P4 |
| In a typical week, on how many days do you do moderate-intensity activities as part of your work? | Number of days └─┘ | P5 |
| How much time do you spend doing moderate-intensity activities at work on a typical day? | Hours : minutes └─┴─┘ : └─┴─┘<br>hrs mins | P6 (a-b) |
| **Travel to and from places** | | |
| The next questions exclude the physical activities at work that you have already mentioned.<br>Now I would like to ask you about the usual way you travel to and from places. For example to work, for shopping, to market, to place of worship. *[Insert other examples if needed]* | | |
| Do you walk or use a bicycle *(pedal cycle)* for at least 10 minutes continuously to get to and from places? | Yes 1<br>No 2 *If No, go to P 10* | P7 |
| In a typical week, on how many days do you walk or bicycle for at least 10 minutes continuously to get to and from places? | Number of days └─┘ | P8 |
| How much time do you spend walking or bicycling for travel on a typical day? | Hours : minutes └─┴─┘ : └─┴─┘<br>hrs mins | P9 (a-b) |

**Table S3.** Stratification of Baseline Data by Survey Completion at Year 1.

| | AD ($n$ = 36) | Non-AD ($n$ = 52) | ANOVA $p$-value |
|---|---|---|---|
| **BMI** | 27.6 (SD = 5.08) | 31.0 (SD = 8.35) | 0.416 |
| Completed T1 | 27.5 (SD = 4.86) | 31.8 (SD = 8.99) | |
| Did not complete T1 | 27.4 (SD = 5.63) | 29.6 (SD = 6.83) | |
| **WHR** | 0.84 (SD = 0.09) | 0.87 (SD = 0.10) | 0.301 |
| Completed T1 | 0.82 (SD = 0.08) | 0.86 (SD = 0.09) | |
| Did not complete T1 | 0.85 (SD = 0.10) | 0.88 (SD = 0.12) | |
| **Age** | 34.9 (SD = 14.50) | 41.8 (SD = 13.20) | 0.588 |
| Completed T1 | 35.1 (SD = 12.21) | 42.3 (SD = 10.61) | |
| Did not complete T1 | 34.8 (SD = 17.90) | 40.6 (SD = 18.27) | |
| **Gender (% Men)** | 19.4% ($n$ = 7) | 23.1% ($n$ = 12) | |
| **Work vigorous activity (%)** | 31.4% ($n$ = 11) | 28.6% ($n$ = 14) | |
| **Mean min per week** | 513 (SD = 1126.7) | 467(SD = 995.7) | 0.257 |
| Completed T1 | 424 (SD = 1164.5) | 373 (SD = 715.2) | |
| Did not complete T1 | 651 (SD = 1092.5) | 680 (SD = 1457.3) | |
| **Work moderate activity (%)** | 65.7% ($n$ = 23) | 44.9% ($n$ = 22) | |
| **Mean min per week** | 887 (SD = 1065.6) | 749 (SD = 1136.7) | 0.182 |
| Completed T1 | 891 (SD = 1141.6) | 564 (SD = 890.9) | |
| Did not complete T1 | 882 (SD = 967.9) | 1168 (SD = 1513.8) | |
| **Walk/cycle for travel (%)** | 88.5% ($n$ = 31) | 77.6% ($n$ = 38) | |
| **Mean min per week** | 717 (SD = 843.9) | 450 (SD = 1097.8) | 0.852 |
| Completed T1 | 643 (SD = 883.8) | 533 (SD = 1297.3) | |
| Did not complete T1 | 843 (SD = 789.7) | 263 (SD = 332.3) | |
| **Vigorous rec activity (%)** | 54.2% ($n$ = 19) | 33.3% ($n$ = 6) | |
| **Mean min per week** | 202 (SD = 312.8) | 113 (SD = 241.9) | 0.401 |
| Completed T1 | 138 (SD = 211.0) | 129 (SD = 274.4) | |
| Did not complete T1 | 312 (SD = 422.8) | 78 (SD = 149.5) | |
| **Moderate rec activity (%)** | 50% ($n$ = 18) | 43.7% ($n$ = 21) | |
| **Mean min per week** | 218 (SD = 410.2) | 102 (SD = 195.7) | 0.857 |
| Completed T1 | 254 (SD = 479.4) | 90 (SD = 170.9) | |
| Did not complete T1 | 157 (SD = 261.0) | 130 (SD = 246.3) | |
| **Daily sitting time (min)** | 272 (SD = 203.8) | 245 (SD = 196.2) | 0.956 |
| Completed T1 | 299 (SD = 222.8) | 225 (SD = 153.0) | |
| Did not complete T1 | 227 (SD = 165.4) | 283 (SD = 270.7) | |
| **Steps per day** | 4573 (SD = 3038.7) | 5388 (SD = 3378.3) | 0.018 |
| Completed T1 | 3792 (SD = 3175.2) | 4404 (SD = 2972.3) | |
| Did not complete T1 | 6524 (SD = 1631.5) | 9327 (SD = 1448.9) | |
| **Stair flights per day** | 5.0 (SD = 2.6) | 9.4 (SD = 5.3) | 0.014 |
| Completed T1 | 4.2 (SD = 2.5) | 7.5 (SD = 3.9) | |
| Did not complete T1 | 7.3 (SD = 1.5) | 17 (SD = 1.4) | |
